# Circular RNA circFNDC3AL Upregulates BCL9 Expression to Promote Chicken Skeletal Muscle Satellite Cells Proliferation and Differentiation by Binding to miR-204

**DOI:** 10.3389/fcell.2021.736749

**Published:** 2021-10-01

**Authors:** Yuanhang Wei, Yongtong Tian, Xinyan Li, Felix Kwame Amevor, Xiaoxu Shen, Jing Zhao, Xiyu Zhao, Xinyi Zhang, Wenling Huang, Jihong Hu, Jie Yi, Lei Yan, Yao Zhang, Diyan Li, Menggen Ma, Qing Zhu, Huadong Yin

**Affiliations:** ^1^Farm Animal Genetic Resources Exploration and Innovation Key Laboratory of Sichuan Province, Sichuan Agricultural University, Chengdu, China; ^2^College of Resources, Sichuan Agricultural University, Chengdu, China

**Keywords:** myogenesis, circFNDC3AL, SMSCs, miR-204, BCL9, chicken

## Abstract

Skeletal muscle is a heterogeneous tissue that is essential for initiating movement and maintaining homeostasis. The genesis of skeletal muscle is an integrative process that lasts from embryonic development to postnatal stages, which is carried out under the modulation of many factors. Recent studies have shown that circular RNAs (circRNAs), a class of non-coding RNAs, are involved in myogenesis. However, more circRNAs and their mechanisms that may regulate skeletal muscle development remain to be explored. Through in-depth analysis of our previous RNA-Seq data, circFNDC3AL was found to be a potentially functional circRNA highly expressed during embryonic development of chicken skeletal muscle. Therefore, in this study, we investigated the effect of circFNDC3AL on skeletal muscle development in chickens and found that circFNDC3AL promoted chicken skeletal muscle satellite cell (SMSC) proliferation and differentiation. To gain a thorough understanding of the exact modulatory mechanisms of circFNDC3AL in chicken skeletal muscle development, we performed target miRNA analysis of circFNDC3AL and found that circFNDC3AL has a binding site for miR-204. Subsequently, we demonstrated that miR-204 inhibited chicken SMSC proliferation and differentiation, which showed the opposite functions of circFNDC3AL. Furthermore, we identified the miR-204 target gene B-cell CLL/lymphoma 9 (BCL9) and validated that miR-204 had an inhibitory effect on BCL9, while the negative effect could be relieved by circFNDC3AL. In addition, we verified that BCL9 performed the same positive functions on chicken SMSC proliferation and differentiation as circFNDC3AL, as opposed to miR-204. In conclusion, our study identified a circRNA circFNDC3AL that upregulates BCL9 expression to promote the proliferation and differentiation of chicken SMSCs by binding to miR-204.

## Introduction

Skeletal muscle is the largest tissue in the body of adult animals and is a crucial organ for initiating movements and maintaining homeostasis. The healthy development of skeletal muscle is important for the health of individual animals, otherwise it will lead to diseases such as Duchenne muscular dystrophy (DMD), which has been generally studied (Jin and Jeong, 2015). Skeletal muscle development is closely related to the activity of myogenic stem cells. The proliferation and differentiation of SMSCs give rise to the fusion of new myotubes and ultimately contribute to the formation of new myofibers (Das et al., 2019). Myogenesis is a complicated process that depends on the precise spatiotemporal expression of regulatory factors (Zhang et al., 2019). With the development of bioinformatics, increasing transcripts and non-coding RNAs have been found to be involved in modulating skeletal muscle growth (Cesana et al., 2011; Peng et al., 2019).

As a class of endogenous small non-coding RNAs, micro RNAs (miRNAs) commonly suppress the translation of mRNAs by binding to the 3′ untranslated region (3′ UTR), and further regulate the expression levels of target genes (Ambros, 2001). miRNAs have been studied in various biological processes, such as skeletal muscle development (Bartel, 2004; Mcdaneld et al., 2009). Previous studies have shown that miR-204 inhibits C2C12 differentiation and represses mouse skeletal muscle regeneration (Cheng et al., 2018; Tan et al., 2020). However, the regulatory effect of miR-204 on the development of chicken skeletal muscles remains to be explored.

Circular RNAs (circRNAs) have unique covalently closed loop structure without 5′ or 3′ polarity (Agnieszka et al., 2015; Chen and Yang, 2015). Emerging evidence indicates that circRNAs play an important role in gene expression regulation since they have been broadly identified in eukaryotes (Jeck et al., 2013; Hansen et al., 2014). For instance, circRNAs might serve as miRNA sponges (Hansen et al., 2013), possess the capacity for translation (Legnini et al., 2017), and regulate the splicing of parent linear mRNAs (Zhang et al., 2013). With the continuous exploration of its diverse functions, circRNAs have been shown to regulate various biological processes extensively, including skeletal muscle development (Ouyang et al., 2018b; Yue et al., 2020).

In our previous study, 24 embryonic chicken breast muscles were sequenced, and a large number of circRNAs differentially expressed between broilers and layers were screened out (Shen et al., 2019). Previous study sequenced the circRNAs those were expressed from embryonic stage to 1 day post hatch in Xinghua chickens, authors established three groups (E11, E16 and P1) and analyzed the differentially expressed circRNAs among these three time points (Ouyang et al., 2018b). Referring to their screening method, we re-analyzed our sequencing data (SRA database accession number: PRJNA516545), in order to explore the differentially expressed circRNAs in broilers at four different embryonic time points (E10, E13, E16, and E19). After the process, 431 differentially expressed circRNAs were identified (Supplementary Table S1). A circRNA generated from a fibronectin type III domain containing 3A-like gene (circFNDC3AL) showed a relatively high expression level. We found the expression of circFNDC3AL gradually increased during embryonic development of chicken skeletal muscle, suggesting that circFNDC3AL might be functional in myogenesis. Thus, in this study, we demonstrated the effects of circFNDC3AL on chicken SMSC proliferation and differentiation and explored the potential mechanism.

## Materials and Methods

### Ethics Statement

This study was approved by the Animal Welfare Committee of Sichuan Agricultural University, by which all the animal experiments were directed and supervised (Approval number 2019202010).

### Sample Collecting

The ROSS 308 broilers and fertilized eggs were supplied by Sichuan Zhengda Food Co., Ltd (Suining, China). All the eggs were incubated at 37.8°C with 60 ± 10 % humidity using an Automatic Incubator (Oscilla, Shandong, China). We collected heart, liver, spleen, lung, kidney, skeletal muscle, brain, intestine, and adipose from three chickens. In addition, breast muscles were collected from four embryonic stages (E10, E13, E16, E19) with three replicates each. The tissue samples were immediately stored in −80°C for further analyses.

### CircFNDC3AL Validation

To identify circFNDC3AL, Sanger sequencing and RNase R digestion were performed. CircFNDC3AL was amplified by divergent primers, and the resulting PCR products were purified by gel electrophoresis, and sent for Sanger sequencing at Sangon Biotech Co. Ltd. (Shanghai, China). For RNase R treatment, 1 μg of total RNA was incubated with 1 μL RNase R at 37°C for 10 min, then inactivated RNase R at 95°C for 10 min and stored at 4°C. The treated RNAs were reverse transcribed into cDNA for qPCR.

### RNA Oligonucleotides and Vector Construction

We prepared three circFNDC3AL siRNAs, three BCL9 siRNAs and their corresponding NC siRNAs. All these siRNAs, miR-204 inhibitors or mimics were synthesized by GenePharma Co. Ltd. (Shanghai, China). For the overexpression vector construction (pCD2.1-circFNDC3AL), we used the KpnI and BamHI (TaKaRa, Dalian, China) restriction sites to construct the linear sequence of circFNDC3AL into the pCD2.1-ciR vector (Geneseed Biotech, Guangzhou, China). All RNA oligonucleotides used in this study are listed in Table 1.

**TABLE 1:** RNA oligonucleotides in this study.

**Name**	**Sequences (5′-3′)**	**Modification**
circFNDC3AL siRNA 1	GGUUCUUCCACUGCAAACUTT	
	AGUUUGCAGUGGAAGAACCTT	
circFNDC3AL siRNA 2	GAGAACGAGGUUCUUCCACTT	
	GUGGAAGAACCUCGUUCUCTT	
circFNDC3AL siRNA 3	GCAGUGGAAGAACCUCGUUTT	
	AACGAGGUUCUUCCACTGCTT	
BCL9 siRNA 1	CUCCAUGACACCUUCAAAUTT	
	AUUUGAAGGUGUCAUGGAGTT	
BCL9 siRNA 2	CCCACCAGUAACCCUAAUATT	
	UAUUAGGGUUACUGGUGGGTT	
BCL9 siRNA 3	GGGCAUUAAUUCUCAGAAUTT	
	AUUCUGAGAAUUAAUGCCCTT	
siRNA NC	UUCUCCGAACGUGUCACGUTT	
	ACGUGACACGUUCGGAGAATT	
miR-204 inhibitor	AGGCAUAGGAUGACAAAGGGAA	2′ OMe
inhibitor NC	CAGUACUUUUGUGUAGUACAA	
miR-204 mimics	UUCCCUUUGUCAUCCUAUGCCU	
mimics NC	UUGUACUACACAAAAGUACUG	

### Cell Culture and Treatment

Chicken SMSCs were isolated from the breast muscle of ROSS 308 broiler chickens at 4-7 days post hatching. The breast muscles were cut to approximately 1 mm^3^ and digested with collagenase Type I (Gibco, Langley, OK, United States) and 0.25% trypsin (Gibco). After filtration and resuspension, the cells were seeded into culture dishes, and then SMSCs were obtained by differential attachment. We prepared growth medium [GM: Dulbecco’s modified Eagle’s medium (DMEM) (Gibco) + 10% fetal bovine serum (Gibco)] and differentiation medium [DM: DMEM + 2% horse serum (Gibco)] as previously described (Yin et al., 2020). For the proliferation studies, SMSCs were cultured in GM and transfected using Lipofectamine 3,000 (Invitrogen, Carlsbad, California, United States) according to the manufacturer’s instructions when the cell confluence reached approximately 50%. For differentiation studies, SMSCs were firstly cultured in GM until the confluence approached approximately 90%, then SMSCs were transfected and cultured in DM. DF-1 cells were specifically cultured in GM for luciferase reporter assays. Medium was refreshed every 24 h.

### RNA Isolation, cDNA Synthesis, and Quantitative Real-Time PCR (qPCR)

Total RNA isolation and cDNA synthesis were performed according to the manufacturer’s instructions as previously reported (Han et al., 2019). qPCR was performed using a CFX96-Touch^TM^ real-time PCR detection system (Bio-Rad, Hercules, CA, United States). Three biological replicates were set for each sample, and the results were determined using the 2^–ΔΔ*Ct*^ method. The internal control gene β-actin, GAPDH and U6 (for miRNA) was used in this study. The primers used in this study are listed in Table 2.

**TABLE 2:** Primers used for the qPCR.

**Gene**	**Primer sequence (5′-3′)**	**Product length (nt)**
circFNDC3AL	F: AGTTGTGAGCCAGATTGT	226
	R: GTCCTCCATCTTCACCACATAAAGT	
MYOD	F: GCCGCCGATGACTTCTATGA	66
	R: CAGGTCCTCGAAGAAGTGCAT	
MYOG	F: CGTGTGCCACAGCCAATG	63
	R: CCGCCGGAGAGAGACCTT	
MYHC	F: GAAGGAGACCTCAACGAGATGG	138
	R: ATTCAGGTGTCCCAAGTCATCC	
CCND1	F: CTCCTATCAATGCCTCACA	165
	R: TCTGCTTCGTCCTCTACA	
CCND2	F: GCACAACTTACTGACGATAG	125
	R: CTTCACAGACCTCCAACAT	
PCNA	F: AACACTCAGAGCAGAAGAC	225
	R: GCACAGGAGATGACAACA	
CDK2	F: CCAGAACCTCCTCATCAAC	171
	R: CAGATGTCCACAGCAGTC	
β-actin	F: GTCCACCGCAAATGCTTCTAA	78
	R: TGCGCATTTATGGGTTTTGTT	
GAPDH	F: CCAGAACATCATCCCAGCGTC	136
	R: ACGGCAGGTCAGGTCAACAA	
miR-204	F: TTCCCTTTGTCATCCTATGCCT	/
	R: CAGGTCCAGTTTTTTTTTTTTTT	
U6	F: GGGCCATGCTAATCTTCTCTGTA	/
	R: CAGGTCCAGTTTTTTTTTTTTTT	

### EdU Assay

Cell-light EdU Apollo567 In Vitro Kits (RiboBio, Guangzhou, China) was used to perform EdU assays. After 48 h of transfection, the GM was replaced with the medium prepared with EdU reagent and incubated for 3 h. Then the cells were fixed and permeated before they were stained with Hoechst 33,342. Each treatment had 6 replicates. The images were captured using a fluorescence microscope (Olympus, Japan) and the results were determined using the software Image-Pro Plus.

### Cell Counting Kit 8 (CCK-8) Assay

A cell Counting Kit-8 kit (Multisciences, Hangzhou, China) was used to perform the CCK-8 assays. After transfection for 12 h, 24 h, 36 h, and 48 h, each well of SMSCs was incubated with 10 μL of CCK-8 reagent for 1 h. Each treatment had 8 replicates. The absorbance of SMSCs at 450 nm was measured using a Microplate Reader (Thermo Fisher, Carlsbad, CA, United States).

### Flow Cytometric Cell Cycle Analysis

The cells were collected after 48 h of transfection and fixed with 70% ethanol, overnight at 4°C. SMSCs were incubated with PI/RNase Staining Buffer Solution (BD Biosciences, Franklin Lakes, United States) at 37°C for 15 min before the analyses were conducted. A BD AccuriC6 flow cytometer (BD Biosciences, San Jose, CA, United States) and Modfit software were used. Each treatment was replicated 3 times.

### Western Blot Assay

After 72 h of transfection, SMSCs were collected using a Total Protein Extraction Kit (BestBio, Shanghai, China). Protein concentrations were determined using a bicinchoninic acid (BCA) protein assay kit (BestBio). The samples were separated by SDS-PAGE and then transferred to polyvinylidene fluoride (PVDF) membranes (Millipore Corporation, Billerica, MA, United States). Subsequently, the membranes were blocked using a Quickblock solution (Beyotime, Shanghai, China) for 1 h at room temperature. The membranes were incubated with specific primary antibodies, including anti-MyoG (Biorbyt, Cambridge, United Kingdom; diluted 1: 500), and anti-β-tubulin (Zenbio, Chengdu, China, 1: 1000) at 4°C overnight. The next day, PVDF membranes with proteins were incubated with a specific secondary antibody [anti-mouse/rabbit immunoglobulin G (IgG)] (ZenBio, 1:2000). Finally, the enhanced chemiluminescence (ECL) luminous fluid (Beyotime) was used for detection. The relative gray scales of MyoG were detected using the ImageJ software, and three independent replicates were set for each treatment (National Health Institute, Bethesda, MD, United States).

### Immunofluorescence Assay

After 72 h of transfection, the DM of SMSCs was removed, and the cells were washed with PBS (Gibco) 3 times before SMSCs were fixed with 4% paraformaldehyde for 30 min. Subsequently, 0.1% Triton X-100 was used to permeate the SMSCs. The 5% goat serum (Beyotime) was used to block the SMSCs so that the cells could better bind to the primary antibody of Myosin (Santa Cruz, United States; 1: 200). SMSCs were incubated with Rhodamine (TRITC) AffiniPure Goat Anti-Mouse Immunoglobulin G (IgG) (ZenBio; 1: 1000) at 37°C for 1 h. Then, DAPI (Beyotime; 1: 50) was used to stain the cell nuclei before the required images were captured. The results were determined using Image-Pro Plus software.

### Target miRNAs and Genes Prediction and Luciferase Reporter Assay

RNAhybrid software^1^ was used to predict the relationship between miRNAs and circRNAs or target genes. TargetScan^2^, miRDB^3^ and Diana Tools^4^ were used to predict the target genes of specific miRNAs. KEGG^5^ was used to analyze the potential functions for the target genes of miR-204. The pmirGLO-circFNDC3AL-WT, pmirGLO-circFNDC3AL-MT, pmirGLO-BCL9-WT, and pmirGLO-BCL9-MT were synthesized by Sangon Biotech (Shanghai, China). DF-1 cells seeded in 48 wells plates were transfected with pmirGLO-WT/MT, miR-204 mimics, and mimics NC (GenePharma). After 48 h of incubation, DF-1 cells were washed with PBS (Gibco). Subsequently, the cells were lysed with Lysis Buffer (10^*x*^; GeneCopoeia, Rockville, MD, United States) at room temperature for 20 min. Then, the cells were successively incubated with 1^*x*^ Luc Buffer I and 1^*x*^ Luc Buffer. Ultimately, an fluorescence/multi-detection microplate reader (Biotek, Shoreline, WA, United States) was used to detect the luminescent values of firefly and Renilla luciferases.

### Statistical Analysis

For statistical analysis, each experiment was performed in triplicate. Unpaired Student’s *t*-test was used to analyze the results of two groups, whereas one-way ANOVA and Tukey’s test analyses were performed to compare differences among groups. All statistical analyses were performed using SPSS 20.0 (SPSS Inc., Chicago, IL, United States). The results were presented as means ± standard error (SEM). ^∗^ *P* < 0.05, ^∗∗^ *P* < 0.01, and a, b *P* < 0.05 were set as statistical significance.

## Results

### Identification and Expression Profile of CircFNDC3AL

**FIGURE 1:**
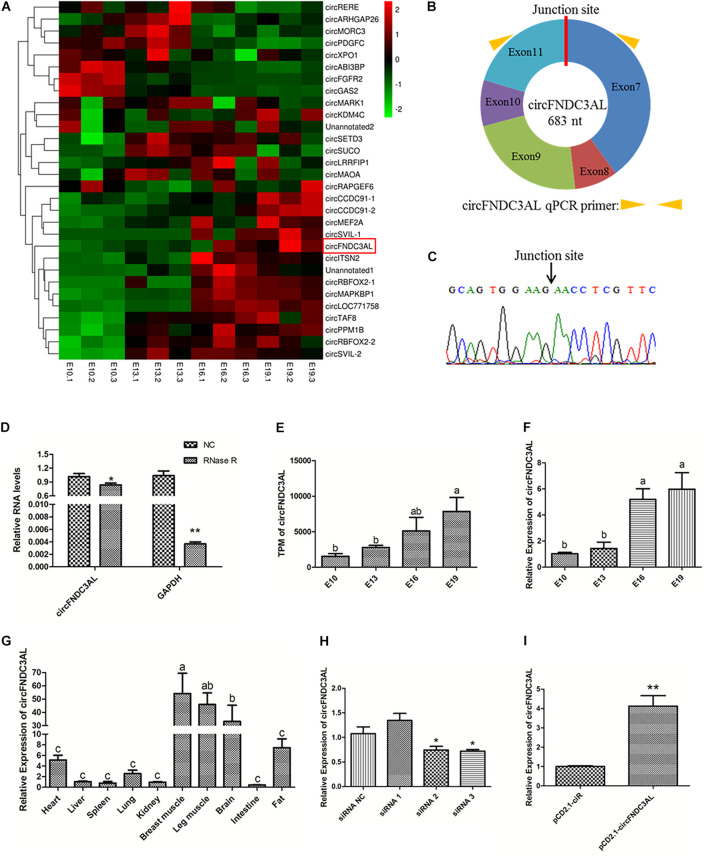
Validation and expression pattern of circFNDC3AL in chicken. **(A)** Heatmap of the top 30 highly and differentially expressed circRNAs. **(B)** Schematic diagram of circFNDC3AL. **(C)** Sanger sequencing investigated the back-splicing junction site of the circFNDC3AL. **(D)** Relative expressions of circFNDC3AL and GAPDH treated with RNase R. **(E)** Expression levels of circFNDC3AL at E10-E19 four time points obtained by RNA-seq. **(F)** Expression levels of circFNDC3AL at E10-E19 four time points determined by qPCR. **(G)** CircFNDC3AL abundance in different tissues of 3 ROSS-308 broilers. **(H,I)** circFNDC3AL expression abundance in SMSCs with three siRNAs or siRNA NC, pCD2.1-circFNDC3AL or pCD2.1-ciR were transfected. Biological statistics analysis was conducted using the One-way ANOVA **(C,D)** and Student’s *t*-test **(E,F)** *P < 0.05; **P < 0.01; a, b *P* < 0.05.

By reanalyzing our previous sequencing data, we found that circFNDC3AL was differentially expressed between E10 and E19, E13 and E19 (Figure 1A). Bioinformatics analysis showed that circFNDC3AL is derived from the 7-11 exons of its parent gene FNDC3AL on chicken chromosome 4 (Figure 1B). What’s more, circFNDC3AL showed high expression levels in the circRNA transcriptome of broiler breast muscle (ranking of expression level: 27/4,226). To validate the closed loop structure of circFNDC3AL, we performed Sanger sequencing and successfully detected the junction site of circFNDC3AL (Figure 1C). Meanwhile, we found that circFNDC3AL showed a stronger resistance to RNase R than the linear internal reference gene (GAPDH) (*P* < 0.05; Figure 1D). This evidence confirms the existence of circFNDC3AL.

CircFNDC3AL was detected to increase with the embryonic development of chicken skeletal muscle (*P* < 0.05; Figure 1E). To better understand the expression profile of circFNDC3AL, we investigated and found that the expression level of circFNDC3AL significantly increased at four time points during the embryonic stage by qPCR (*P* < 0.05; Figure 1F). The qPCR results confirmed the accuracy of our previous sequencing data. Moreover, circFNDC3AL was found to be enriched in chicken skeletal muscles, and its abundance was significantly higher than that in other tissues (*P* < 0.05; Figure 1G). These results confirmed that circFNDC3AL is a novel circular RNA that might be associated with chicken skeletal muscle development.

### Validation of CircFNDC3AL Interference and Overexpression Efficiency

Three circFNDC3AL siRNAs and an overexpression vector were synthesized and transfected into chicken SMSCs. We determined circFNDC3AL expression levels in each treatment group and compared them with those in the corresponding NC groups. Eventually, siRNA 3 presented the best interference efficiency among the three siRNAs (*P* < 0.05; Figure 1H). Meanwhile, pCD2.1-circFNDC3AL also improved the expression level of circFNDC3AL compared to that of pCD2.1-ciR (*P* < 0.01; Figure 1I). Hence, circFNDC3AL siRNA 3 and pCD2.1-circFNDC3AL were selected for further verification.

### CircFNDC3AL Promotes the Proliferation of SMSCs in Chicken

**FIGURE 2:**
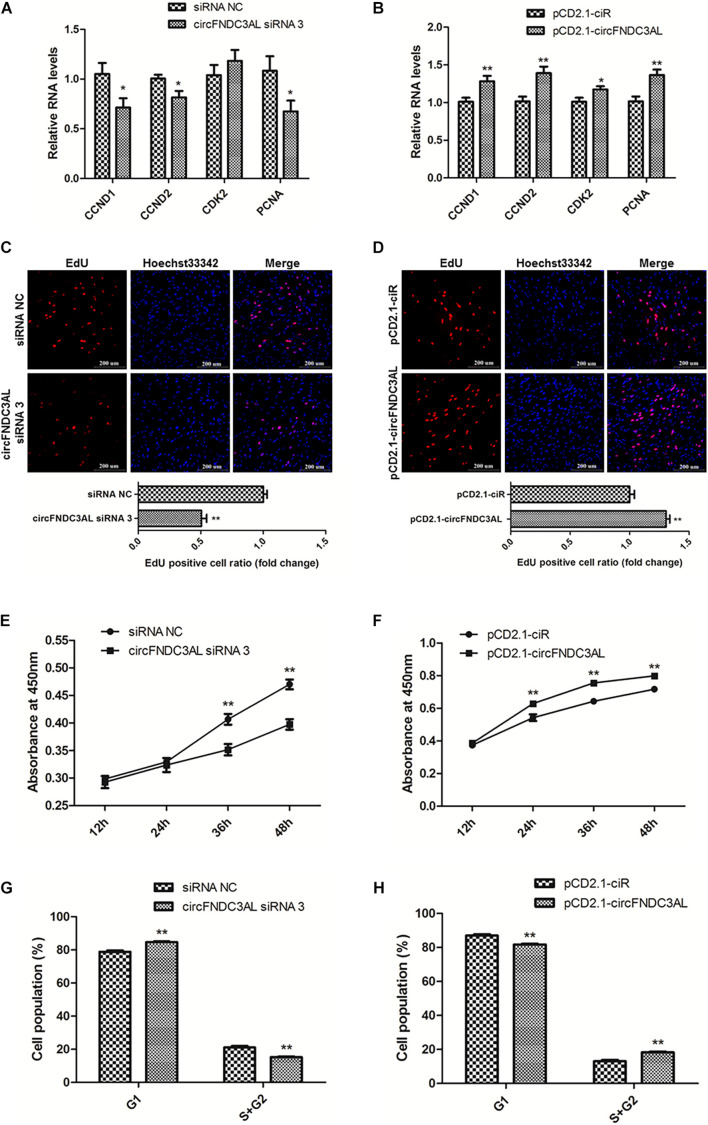
CircFNDC3AL promotes the proliferation of SMSCs in chicken. **(A,B)** Proliferation-related genes mRNA levels were determined using qPCR after SMSCs were transfected with circFNDC3AL siRNA or overexpression plasmid. **(C,D)** EdU assays were conducted after the cells were transfected with circFNDC3AL siRNAs or vectors. **(E,F)** SMSCs absorbance at 450 nm was detected by CCK-8 assay after the cells were transfected with circFNDC3AL siRNAs or vectors. **(G,H)** Cell cycle analysis of SMSCs following circFNDC3AL overexpression or inhibition. The scale bar represents 200 μm. Values represent as means ± S.E.M. of three biological replicates. *P < 0.05; **P < 0.01.

qPCR, EdU, CCK-8, and flow cytometry assays were conducted to explore the effects of circFNDC3AL on SMSC proliferation. The expression levels of proliferation-related genes were determined by qPCR, such as cyclin D1 (CCND1), cyclin D2 (CCND2), cyclin dependent-kinase 2 (CDK2), and proliferating cell nuclear antigen (PCNA). Results showed that knockdown of circFNDC3AL downregulated the expression levels of these genes, on the other hand, circFNDC3AL overexpression promoted these genes expression (*P* < 0.05; Figures 2A,B). Moreover, EdU assays verified that the proliferation of SMSCs was reduced by circFNDC3AL knockdown but increased by cirFNDC3AL overexpression (*P* < 0.01; Figures 2C,D). Meanwhile, CCK-8 assays confirmed the results of EdU assays, and the proliferation of SMSCs was significantly repressed when circFNDC3AL expression was interfered but accelerated when circFNDC3AL was overexpressed (*P* < 0.01; Figures 2E,F). In addition, cell cycle analysis revealed that the proportion of SMSCs in the S and G2 phases was observably decreased by circFNDC3AL knockdown, whereas significantly increased by circFNDC3AL overexpression (*P* < 0.01; Figures 2G,H). These results indicate that circFNDC3AL promotes chicken SMSC proliferation.

### CircFNDC3AL Promotes the Differentiation of SMSCs in Chicken

**FIGURE 3:**
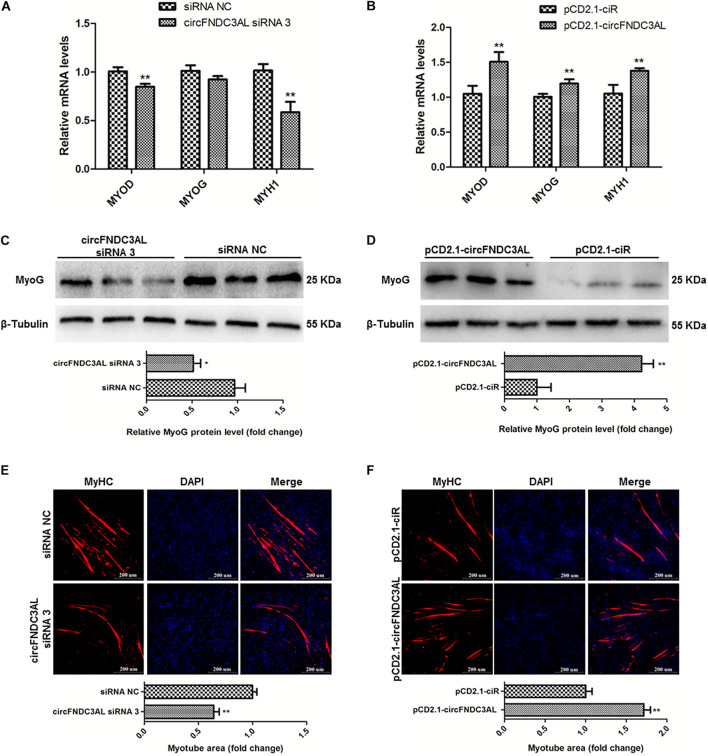
CircFNDC3AL promotes the differentiation of SMSCs in chicken. **(A,B)** Differentiation-related genes mRNA levels were determined using qPCR after SMSCs were transfected with circFNDC3AL siRNA or overexpression plasmid. **(C,D)** MyoG protein levels were detected by western blot after circFNDC3AL were interfered or overexpressed in SMSCs. **(E,F)** Myotube areas were calculated at 72 h after circFNDC3AL was interfered or overexpressed in SMSCs. The scale bar represents 200 μm. Values represent as means ± S.E.M. of three biological replicates. *P < 0.05; **P < 0.01.

qPCR, western blotting, and immunofluorescence assays were performed to investigate the ability of circFNDC3AL to modulate SMSC differentiation. After SMSCs were transfected with circFNDC3AL siRNAs or overexpression vectors, we detected the expression levels of myogenic differentiation 1 (MyoD1), myogenin (MyoG) and myosin heavy chain (MyHC) by qPCR. Results showed that circFNDC3AL knockdown inhibited the expression of these differentiation-related genes, whereas circFNDC3AL overexpression promoted the expression levels of these genes (*P* < 0.01; Figures 3A,B). Through western blot assays, we found that protein levels of MyoG were reduced by circFNDC3AL knockdown but increased by circFNDC3AL overexpression (*P* < 0.05; Figures 3C,D). Furthermore, we conducted immunofluorescence assays and determined that myotube area was relatively downregulated by knockdown of circFNDC3AL but promoted by circFNDC3AL overexpression (*P* < 0.01; Figures 3E,F). These results suggest that circFNDC3AL could promote SMSC differentiation.

### CircFNDC3AL Could Bind miR-204

**FIGURE 4:**
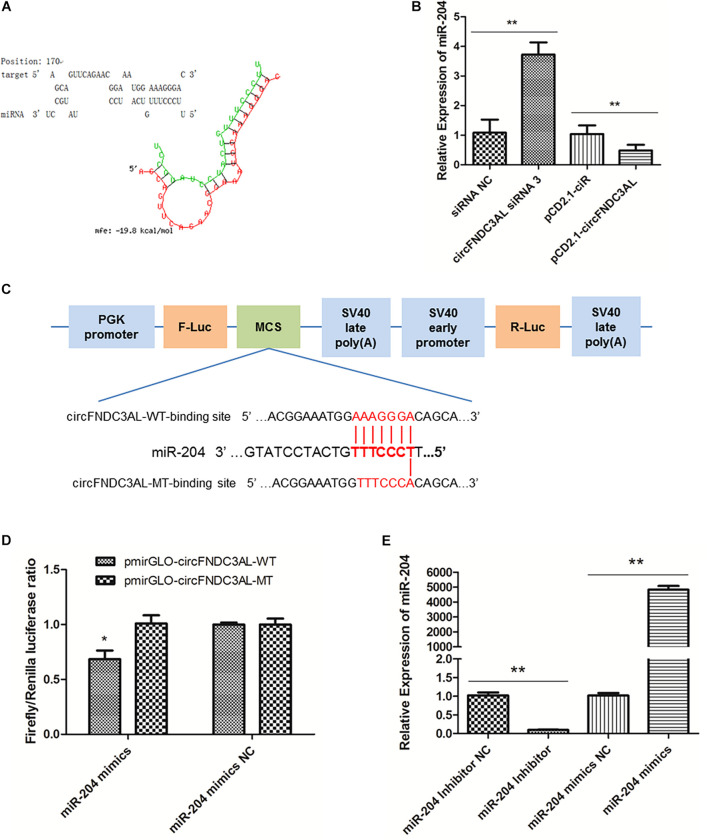
circFNDC3AL could bind with miR-204. **(A)** Target site where miR-204 binds to circFNDC3AL was predicted using RNAhybrid software. **(B)** Expression levels of miR-204 were determined using qPCR after SMSCs were transfected with interfered or overexpressed circFNDC3AL. **(C)** Schematic diagram of wild type or mutant type of miR-204 target site designed for luciferase reporter assays. **(D)** Firefly/Renilla luciferase ratio was detected after DF-1 cells were transfected with pmirGLO-circFNDC3AL-WT/pmirGLO-circFNDC3AL-MT or miR-204 mimics/mimics NC. **(E)** Expression levels of miR-204 were determined using qPCR after SMSCs were transfected with miR-204 inhibitor or mimics. Values represent as means ± S.E.M. of three biological replicates. *P < 0.05; **P < 0.01.

Several miRNAs were successfully predicted as alternative miRNAs targeting circFNDC3AL using RNAhybrid software, and we found that miR-204 had stable binding site for circFNDC3AL (Figure 4A). To further explore the interaction relationship between circFNDC3AL and miR-204, we determined miR-204 expression by qPCR after SMSCs were transfected with circFNDC3AL siRNA3 or overexpression vector. The results indicated that miR-204 expression level was elevated by circFNDC3AL knockdown and decreased by circFNDC3AL overexpression (Figure 4B). Furthermore, we constructed luciferase reporters with wild-type and mutant-type binding sites of circFNDC3AL and miR-204. For wild-type reporter, miR-204 mimics could restrict the expression of Firefly luciferase (F-Luc). This suggests binding to the complementary sequence derived from circFNDC3AL cloned into the Multiple cloning site (MCS). Expression of downstream Renilla luciferase (R-Luc) was not affected. Conversely, miRNAs could not affect the expression of both F-Luc and R-Luc in the mutant-type reporter (Figure 4C). Therefore, we could validate the target relationship between circFNDC3AL and miR-204 by detecting luciferase ratio of F-Luc and R-Luc. Luciferase reporter assays showed that the firefly luciferase activity of pmirGLO-circFNDC3AL-WT was significantly reduced compared to that of pmirGLO-circFNDC3AL-MT after co-transfection with miR-204 mimics into DF-1 cells (*P* < 0.05; Figure 4D). Thus, we hypothesized that circFNDC3AL targets with miR-204.

### MiR-204 Represses the Proliferation of SMSCs in Chicken

**FIGURE 5:**
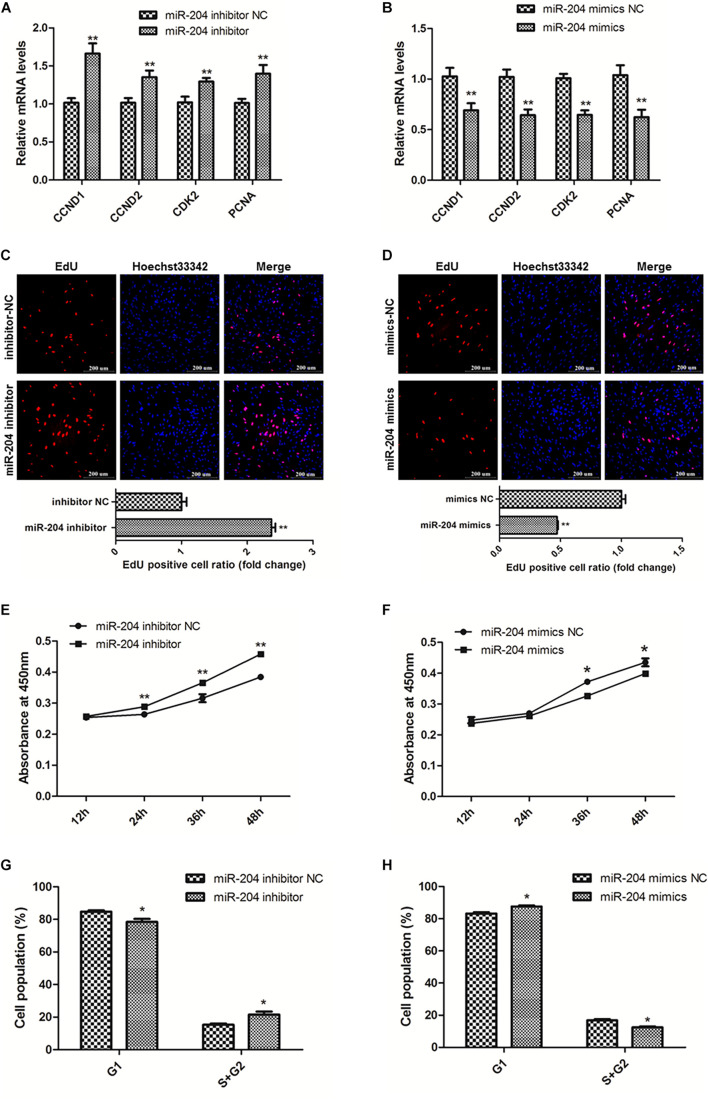
MiR-204 represses the proliferation of SMSCs in chicken. **(A,B)** Proliferation-related genes mRNA levels were determined using qPCR after SMSCs were transfected with miR-204 inhibitor or mimics. **(C,D)** EdU assays were conducted after the cells were transfected with miR-204 inhibitor or mimics. **(E,F)** SMSCs absorbance at 450 nm was detected by CCK-8 assay after the cells were transfected with miR-204 inhibitors or mimics. **(G,H)** Cell cycle analysis of SMSCs following miR-204 overexpression or inhibition. The scale bar represents 200 μm. Values represent as means ± S.E.M. of three biological replicates. *P < 0.05; **P < 0.01.

To explore the role of miR-204 in SMSC development, we used miR-204 mimics or inhibitors, as well as the respective negative control, to regulate the expression of miR-204 in SMSCs. Through qPCR we determined that miR-204 expression could be significantly reduced by miR-204 inhibitor, but promoted by miR-204 mimics (*P* < 0.01; Figure 4E). To further understand the effects of miR-204 on SMSC proliferation, we conducted qPCR, EdU, CCK-8, and cell cycle analysis. For qPCR assays, we detected the expression levels of CCND1, CCND2, CDK2, and PCNA. We found that these gene levels were downregulated when SMSCs were transfected with miR-204 mimics, whereas miR-204 inhibitor could facilitate the expression of these genes (*P* < 0.01; Figures 5A,B). Moreover, EdU and CCK-8 assays were in agreement with these results and indicated that miR-204 mimics might inhibit SMSC proliferation, on the other hand, the miR-204 inhibitor promoted the proliferation of SMSCs (*P* < 0.05; Figures 5C-F). In addition, cell cycle analysis revealed that miR-204 mimics effectively prevented SMSCs from entering the S and G2 phases, while the miR-204 inhibitor facilitated the division process of SMSCs (*P* < 0.05; Figures 5G,H). Therefore, we confirmed that miR-204 repressed chicken SMSC proliferation.

### MiR-204 Represses the Differentiation of SMSCs in Chicken

**FIGURE 6:**
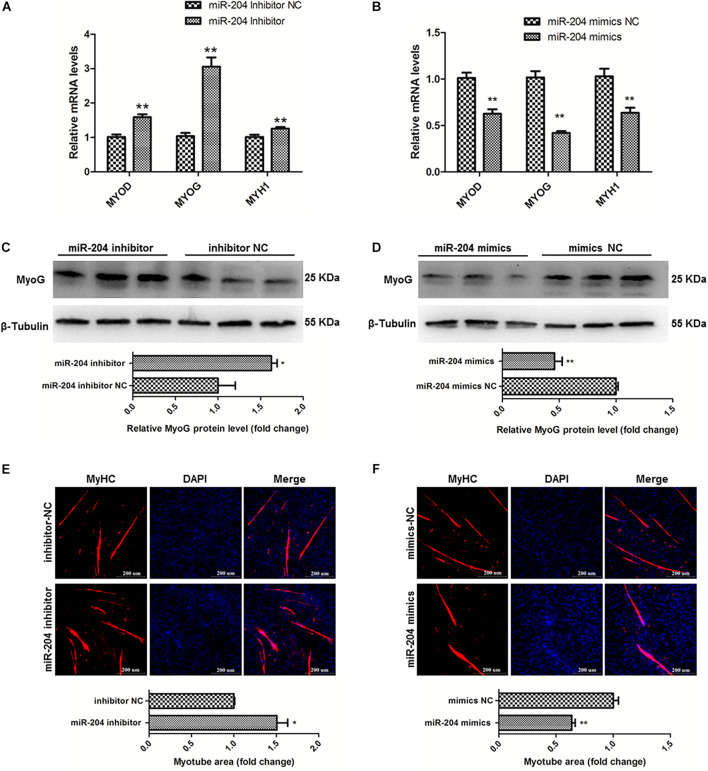
MiR-204 represses the differentiation of SMSCs in chicken. **(A,B)** Differentiation-related genes mRNA levels were determined using qPCR after SMSCs were transfected with miR-204 inhibitor or mimics. **(C,D)** MyoG protein levels were detected by western blot after miR-204 were interfered or overexpressed in SMSCs. **(E,F)** Myotube areas were calculated at 72 h after miR-204 was interfered or overexpressed in SMSCs. The scale bar represents 200 μm. Values represent as means ± S.E.M. of three biological replicates. *P < 0.05; **P < 0.01.

To clarify the effects of miR-204 on chicken SMSC differentiation, we first performed qPCR and found that the expression levels of MyoD1, MyoG and MyHC these three genes were upregulated by miR-204 inhibitor and downregulated by miR-204 mimics (*P* < 0.01; Figures 6A,B). Meanwhile, western blot assays showed that miR-204 inhibitor increased MyoG protein level, which was reduced by miR-204 mimics (*P* < 0.05; Figures 6C,D). Moreover, immunofluorescence of MyHC suggested that myotube formation was facilitated by miR-204 inhibitor, but repressed by miR-204 mimics (*P* < 0.05; Figures 6E,F). Based on these experimental results, we identified the inhibitory effects of miR-204 on chicken SMSC differentiation.

### BCL9 Is a Target Gene of miR-204 and CircFNDC3AL Relieves BCL9 From the Inhibition of miR-204

**FIGURE 7:**
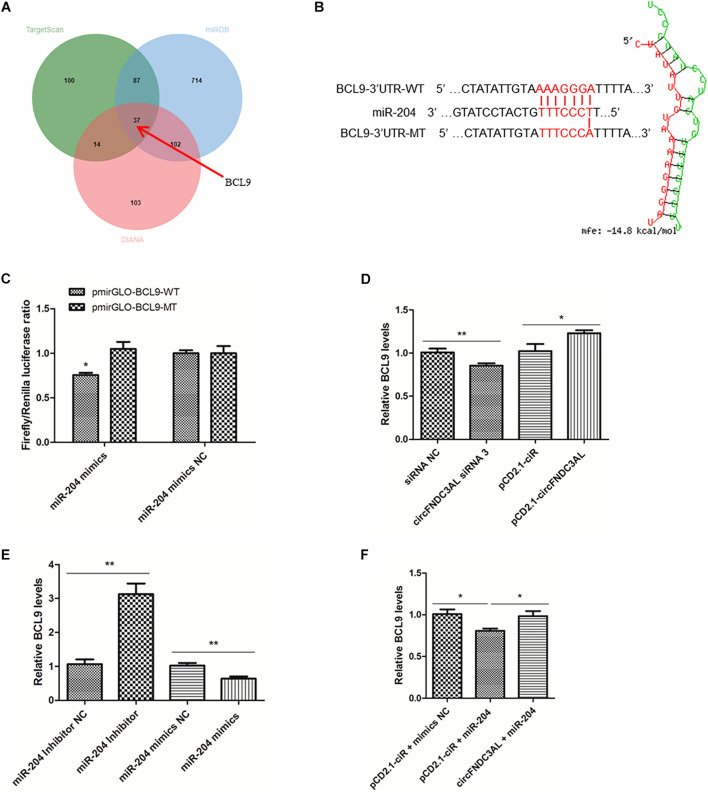
BCL9 is a target gene of miR-204 and circFNDC3AL relieves BCL9 from the inhibition of miR-204. **(A)** The target genes of miR-204 were analyzed using TargetScan, miRDB and Diana softwares. **(B)** Binding site of BCL9 3± S.E.M. of three biological replicates. *P < 0.05; **P < 0.01.

Through the analysis of target mRNAs of miR-204 (Supplementary Table S2), we scanned a group of alternative target genes through TargetScan, miRDB and Diana websites. There are 37 genes simultaneously predicted as the target genes of miR-204 by these three websites (Figure 7A). Combined with the analysis results of KEGG (Supplementary Table S3), we eventually identified BCL9, which is the only gene related to skeletal muscle among the target genes of miR-204 (GO: 0014908∼myotube differentiation involved in skeletal muscle regeneration, GO: 0035914∼skeletal muscle cell differentiation). The binding site of BCL9 3′UTR and miR-204 was predicted using RNAhybrid (Figure 7B). Furthermore, luciferase reporter assays were conducted using DF-1 cells and demonstrated that miR-204 could only bind to the wild type reporter of BCL9, but not to the mutant reporter (*P* < 0.05; Figure 7C). In addition, SMSCs were cultured in GM to further study the regulation of circFNDC3AL or miR-204 to BCL9. qPCR showed that circFNDC3AL promoted BCL9 expression (*P* < 0.05; Figure 7D), whereas miR-204 inhibited the expression of BCL9 (*P* < 0.01; Figure 7E). Subsequently, circFNDC3AL overexpression vectors and miR-204 mimics were co-transfected into SMSCs to investigate the relative expression of BCL9. Comparing to the group that SMSCs were co-transfected with pCD2.1-ciR and miR-204 mimics NC, the expression of BCL9 was significantly reduced when SMSCs were co-transfected with pCD2.1-ciR and miR-204 mimics. In addition, we found that circFNDC3AL blocked the inhibitory effect of miR-204 on BCL9 expression (*P* < 0.05; Figure 7F). Altogether, these results indicated that circFNDC3AL upregulated the expression of BCL9 by binding miR-204.

### BCL9 Promotes the Proliferation and Differentiation of SMSCs in Chicken

**FIGURE 8:**
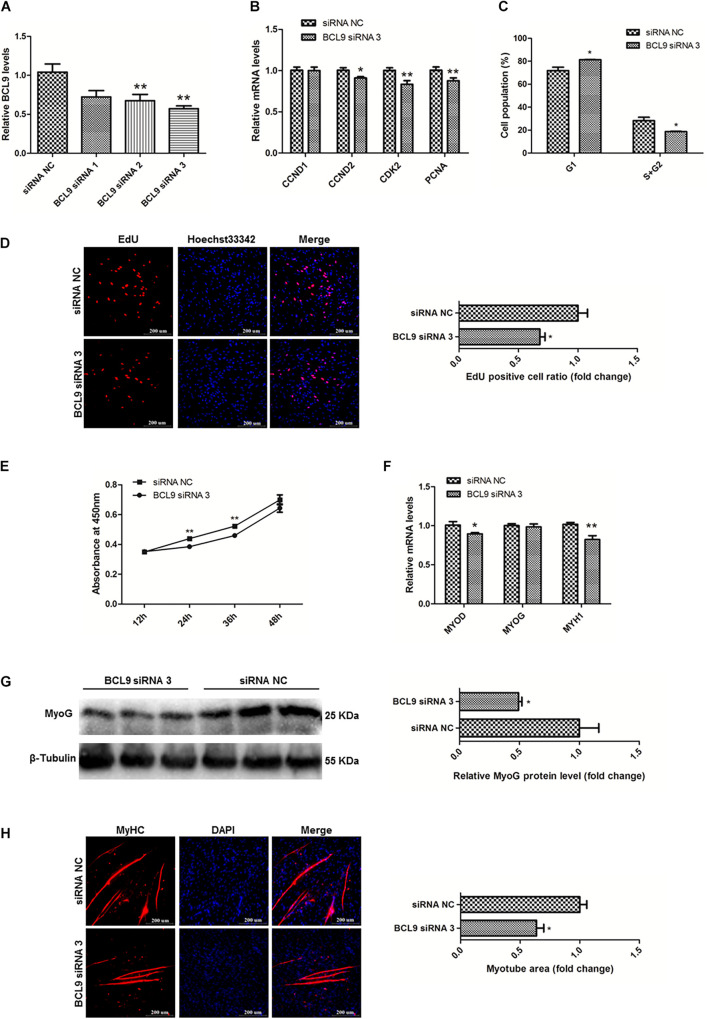
BCL9 promotes the proliferation and differentiation of SMSCs in chicken. **(A)** BCL9 expression levels in SMSCs with three siRNAs or siRNA NC were transfected. **(B)** Proliferation-related genes mRNA levels were determined using qPCR after SMSCs were transfected with interfered BCL9. **(C)** Cell cycle analysis of SMSCs following BCL9 inhibition. **(D)** EdU assays were conducted after the cells were transfected with BCL9 siRNA 3. **(E)** SMSCs absorbance at 450 nm was detected by CCK-8 assay after BCL9 was inhibited. **(F)** Differentiation-related genes mRNA levels were determined using qPCR after SMSCs were transfected with interfered BCL9. **(G)** MyoG protein levels were detected by western blot after BCL9 were interfered in SMSCs. **(H)** Myotube areas were calculated at 72 h after BCL9 was interfered in SMSCs. The scale bar represents 200 μm. Values represent as means ± S.E.M. of three biological replicates. *P < 0.05; **P < 0.01.

To study the functions of BCL9 in myogenesis, we designed three siRNAs to modulate the expression of BCL9 and finally selected BCL9 siRNA 3 (*P* < 0.01; Figure 8A). Then, we carried out qPCR to detect the mRNA levels of the proliferation-related genes CCND1, CCND2, CDK2, PCNA, as before. The results showed that knockdown of BCL9 repressed the expression of these genes in SMSCs in chickens (*P* < 0.05; Figure 8B). Through cell cycle analysis, we found that interference with BCL9 prevented transition of SMSCs from G1 to S and G2 (*P* < 0.05; Figure 8C). EdU assay suggested that BCL9 knockdown repressed SMSC proliferation (*P* < 0.05; Figure 8D), and the CCK-8 assay showed the same result (Figure 8E). On the other hand, to validate the effects of BCL9 on SMSC differentiation in chickens, we performed qPCR, western blot, and immunofluorescence assays. We found that BCL9 knockdown suppressed the expression levels of differentiation-related genes (*P* < 0.05; Figure 8F), as well as the protein level of MyoG (*P* < 0.05; Figure 8G). Furthermore, we found that knockdown of BCL9 reduced myotube formation in SMSCs in chickens (*P* < 0.05; Figure 8H). Our study identified that BCL9 could promote SMSC proliferation and differentiation in chickens, which is similar to the effect of circFNDC3AL while it is opposite to the effect of miR-204.

## Discussion

The modulation for skeletal muscle development has long been focused on protein-coding genes and non-coding RNAs in recent years (Cesana et al., 2011; Li et al., 2017). To date the regulatory effects of protein-coding genes on myogenesis have been extensively studied, and non-coding RNAs are worthy of further exploration. Generally, non-coding RNAs do not have the ability to encode proteins, but previous studies have shown that they can still contain genetic information and have significant functions (Eddy, 2001). As an emerging class of non-coding RNAs, circRNAs are more resistant to RNase R than other linear RNAs, which is benefited from their unique closed loop structure (Agnieszka et al., 2015). With the deepening of research, the functions of circRNAs are constantly updated and expanded, and the regulation of circRNAs on gene expression during myogenesis has also been successively reported. However, the potential functions of circRNAs in the development of skeletal muscles require further study.

The phenotype of an individual organism is commonly determined by genes with genetic information, and the expression of these genes is often regulated by circRNAs (Li et al., 2018). Our previous study performed RNA-seq to explore potential muscle-related circRNAs and screened many of differentially expressed circRNAs between broilers and layers (Shen et al., 2019). In this study, we utilized contrastive combinations between different time points to analyze differentially expressed circRNAs. Ultimately, we screened out circFNDC3AL, which is highly expressed in the breast muscle of broilers and is differentially expressed at multiple time points during fetal development in chickens. Meanwhile, we found that the expression profile of circFNDC3AL is similar to that of circSVIL (Figure 1A), and circSVIL has been proven to be a positive regulator of chicken skeletal muscle development (Ouyang et al., 2018a).

In addition, circFNDC3AL has been identified in chicken intramuscular and abdominal adipose tissue (Zhang et al., 2020), while the expression characteristics and specific functions of circFNDC3AL in skeletal muscle development are still not clear. In this study, we found that circFNDC3AL showed a tissue and developmental stage specific expression pattern, which is an essential feature for most functional circRNAs (Salzman et al., 2013; Venø et al., 2015). We found that circFNDC3AL are enriched in skeletal muscle and its expression level increased along with the fetal development of skeletal muscle. Evidence strongly indicates that circFNDC3AL is a candidate positive regulator of myogenesis in chickens. Furthermore, functional analysis revealed that circFNDC3AL could indeed promote skeletal muscle development, which is similar to that of circFGFR2 (Chen et al., 2018), but contrary to circFGFR4 (Hui et al., 2018).

CircRNAs have been verified to play various functions by serving as miRNA sponges since it was first reported that circRNA CDR1as could bind miR-7 to modulate brain function in zebrafish and mice (Hansen et al., 2013). Subsequently, circRNAs were found to modulate skeletal muscle development in livestock by sponging miRNAs. For instance, circHIPK3 promotes chicken myoblast proliferation and differentiation by sponging miR-30a-3p (Chen et al., 2019), while circLMO7 promotes proliferation but inhibits the differentiation and apoptosis of bovine primary myoblasts by sponging miR-378a-3p (Wei et al., 2017), and circZfp609 represses myogenic differentiation by sponging miR-194-5p to sequester its inhibition on BCLAF1 (Wang et al., 2018). In this study, we predicted that circFNDC3AL has a binding site for miR-204 using RNAhybrid. Subsequently, we verified that circFNDC3AL is combined with miR-204 by performing qPCR and luciferase reporter assays, and found that circFNDC3AL inhibits the expression of miR-204.

Previously, miR-204 has been well studied in human diseases such as acute myeloid leukemia (Aleksandra et al., 2015), and inflammation (Li et al., 2011). Recently, miR-204 has been reported to play a vital role in myogenesis. It has been reported that miR-204 inhibits C2C12 myoblast differentiation by targeting MEF2C and ERRγ (Tan et al., 2020). Moreover, another study revealed that miR-204 could bind Pax7, IGF1, and Mef2c to modulate the process of myogenesis and skeletal muscle regeneration in mouse (Cheng et al., 2018). In this study, we found that miR-204 inhibited chicken SMSC proliferation and differentiation, which is opposite to that of circFNDC3AL.

To gain a better understanding of the molecular regulation mechanism of circFNDC3AL in regulating skeletal muscle development, we further explored the potential downstream target genes for miR-204 using TargetScan, miRDB and Diana. Through the comprehensive analysis of target genes predicted by these three methods, a total of 37 genes (of which, only 33 genes were annotated) were simultaneously predicted to be target genes of miR-204. Subsequently, we used KEGG and found that BCL9 is the only one of these genes that is enriched in skeletal muscle (Supplementary Table S3). Therefore, BCL9 was selected as the target gene of miR-204 in this study. Combined with the results of qPCR and luciferase reporter assays, we confirmed that miR-204 could bind to the 3′UTR of BCL9, which is an essential component of canonical Wnt signaling and mediates skeletal muscle regeneration (Brack et al., 2009). In this study, we confirmed that BCL9 could promote chicken SMSC proliferation and differentiation.

In the development of domestic animal molecular breeding, functional genes are increasingly used to improve the performance of animals such as Myostatin (MSTN) (Aiello et al., 2018), which is a well-studied inhibitor of domestic animal skeletal muscle development. Our results revealed that circFNDC3AL is a positive regulator for the development of skeletal muscle in chickens, suggesting that circFNDC3AL has the potential to be an important target in molecular breeding to improve broiler performance.

## Conclusion

**FIGURE 9:**
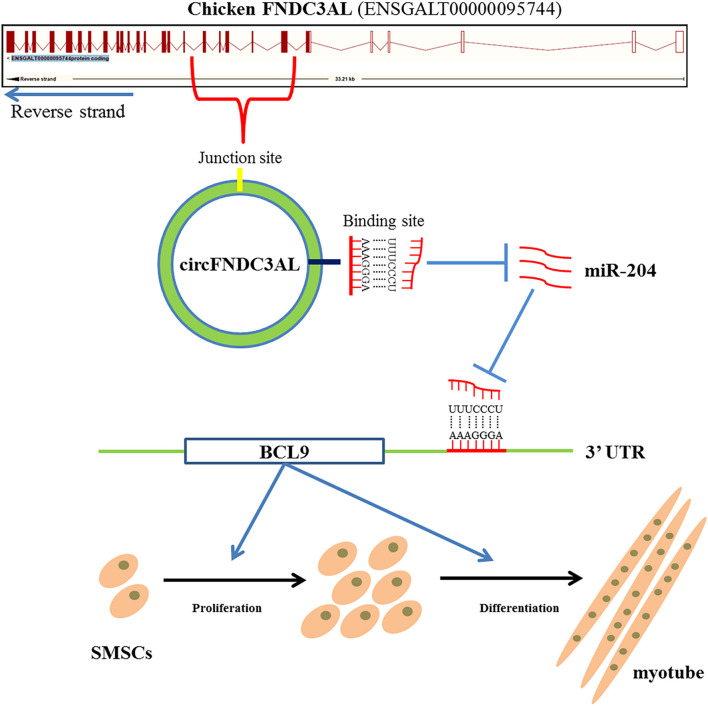
Mechanism of circFNDC3AL regulates chicken SMSC proliferation and differentiation. CircFNDC3AL promotes the proliferation and differentiation of chicken SMSC by up-regulating the expression of BCL9 via binding miR-204.

In conclusion, we confirmed that circular RNA circFNDC3AL upregulates BCL9 expression to promote chicken SMSC proliferation and differentiation by binding miR-204, suggesting that circFNDC3AL may have important significance in improving muscle production in molecular breeding of broilers (Figure 9).

## Data Availability Statement

The datasets presented in this study can be found in online repositories. The names of the repository/repositories and accession number(s) can be found in the article/Supplementary Material.

## Ethics Statement

This study was approved by the Animal Welfare Committee of Sichuan Agricultural University, by which all the animal experiments were directed and supervised (Approval number 2019202010).

## Author Contributions

YW, YT, and HY: conceptualization. YW, YT, XL, XinZ, FA, JY, and LY: formal analysis. HY and QZ: funding acquisition. YW, XS, JZ, XL, XinZ, YT, XiyZ, WH, and JH: investigation. DL: methodology. YZ: project administration; YW, YT, and XL: writing – original draft. HY: writing – review & editing. All authors contributed to the article and approved the submitted version.

## Conflict of Interest

The authors declare that the research was conducted in the absence of any commercial or financial relationships that could be construed as a potential conflict of interest.

## Publisher’s Note

All claims expressed in this article are solely those of the authors and do not necessarily represent those of their affiliated organizations, or those of the publisher, the editors and the reviewers. Any product that may be evaluated in this article, or claim that may be made by its manufacturer, is not guaranteed or endorsed by the publisher.

## Footnotes

1
https://bibiserv.cebitec.uni-bielefeld.de/rnahybrid/

2
http://www.targetscan.org/vert_71/

3
http://mirdb.org/index.html

4
http://diana.imis.athena-innovation.gr/DianaTools/index.php

5
https://www.kegg.jp/
